# Decision tree analysis of genetic risk for clinically heterogeneous Alzheimer’s disease

**DOI:** 10.1186/s12883-015-0304-6

**Published:** 2015-03-28

**Authors:** Jennifer S Yokoyama, Luke W Bonham, Renee L Sears, Eric Klein, Anna Karydas, Joel H Kramer, Bruce L Miller, Giovanni Coppola

**Affiliations:** Memory and Aging Center, Department of Neurology, University of California, San Francisco, CA 94158 USA; Semel Institute for Neuroscience and Human Behavior, Departments of Neurology and Psychiatry, The David Geffen School of Medicine at University of California Los Angeles, Los Angeles, CA 90095 USA

**Keywords:** Alzheimer’s disease, Genetics, Decision tree analysis

## Abstract

**Background:**

Heritability of Alzheimer’s disease (AD) is estimated at 74% and genetic contributors have been widely sought. The ε4 allele of *apolipoprotein E* (*APOE*) remains the strongest common risk factor for AD, with numerous other common variants contributing only modest risk for disease. Variability in clinical presentation of AD, which is typically amnestic (AmnAD) but can less commonly involve visuospatial, language and/or dysexecutive syndromes (atypical or AtAD), further complicates genetic analyses. Taking a multi-locus approach may increase the ability to identify individuals at highest risk for any AD syndrome. In this study, we sought to develop and investigate the utility of a multi-variant genetic risk assessment on a cohort of phenotypically heterogeneous patients with sporadic AD clinical diagnoses.

**Methods:**

We genotyped 75 variants in our cohort and, using a two-staged study design, we developed a 17-marker AD risk score in a Discovery cohort (n = 59 cases, n = 133 controls) then assessed its utility in a second Validation cohort (n = 126 cases, n = 150 controls). We also performed a data-driven decision tree analysis to identify genetic and/or demographic criteria that are most useful for accurately differentiating all AD cases from controls.

**Results:**

We confirmed *APOE* ε4 as a strong risk factor for AD. A 17-marker risk panel predicted AD significantly better than *APOE* genotype alone (P < 0.00001) in the Discovery cohort, but not in the Validation cohort. In decision tree analyses, we found that *APOE* best differentiated cases from controls only in AmnAD but not AtAD. In AtAD, *HFE* SNP rs1799945 was the strongest predictor of disease; variation in *HFE* has previously been implicated in AD risk in non-ε4 carriers.

**Conclusions:**

Our study suggests that *APOE* ε4 remains the best predictor of broad AD risk when compared to multiple other genetic factors with modest effects, that phenotypic heterogeneity in broad AD can complicate simple polygenic risk modeling, and supports the association between *HFE* and AD risk in individuals without *APOE* ε4.

**Electronic supplementary material:**

The online version of this article (doi:10.1186/s12883-015-0304-6) contains supplementary material, which is available to authorized users.

## Background

Alzheimer’s disease (AD) is a devastating neurodegenerative disorder that results in memory impairment and can also involve deterioration of language, visuospatial and/or executive functioning abilities. As the world’s population ages and the number of individuals with AD grows, it will become increasingly important to identify those at highest risk for AD during the earliest stages of—or prior to—disease.

Genetic predictors of AD hold strong potential for identifying those at risk of developing disease. Indeed, a large clinical study will launch in 2015 to assess the utility of AD therapies given to individuals at highest genetic risk for AD but who are still cognitively healthy [1]. These individuals, who carry the ε4 allele of *apolipoprotein E* (*APOE*), have a 2-10x increased risk for developing AD compared to non-carriers [2,3], but not all ε4 carriers go on to develop disease [3,4]. Despite the vast number of genetic studies of AD, which is estimated to be 74% heritable [5], no other common variants have been identified that confer as high a risk as *APOE* ε4. In rare cases, AD is familial, caused by an autosomal dominant mutation in *APP*, *PSEN1*, or *PSEN2* [6,7]. For sporadic late-onset AD (LOAD), numerous common variants of very low effect (odds ratio [OR] ~ 1.1-1.3) have been identified through genome-wide association studies (GWAS) and replicated across multiple large [8], and diverse populations [9,10]. More recently, rare variants (<1% allele frequency) of larger effect size have also been identified as risk conferring (*TREM2* 0.3% [11], *PLD3* < 0.5% [12], *MAPT* 0.3% [13]) or protective against (*APP 0.01%* [14] to 0.62% [15]) AD.

In addition to genetic heterogeneity, there is also clinical heterogeneity in AD. The majority of patients present with amnestic syndromes (AmnAD) but approximately 6-14% of AD patients demonstrate atypical clinical syndromes (AtAD) [16]. These include 1) posterior cortical atrophy (PCA), characterized by predominant visuospatial deficits [17]; 2) the logopenic variant of primary progressive aphasia (lvPPA) [18], characterized by loss in phonologic short-term memory; and 3) dysexecutive/behavioral AD [16] characterized by loss of executive function and/or behavioral changes with retention of memory function.

Genetic and phenotypic heterogeneity strongly support the notion that multiple genetic variants of small effect contribute to disease susceptibility. A multi-locus approach may increase the ability to identify individuals at highest risk for any AD syndrome. The multi-locus approach has had modest success in LOAD, with polygenic risk scoring approaches associating better with LOAD diagnoses and age of onset than *APOE* genotype alone [19-21]. However, most studies have focused on clinically homogeneous groups with primary amnestic presentations.

In this study, we investigated two different strategies for polygenic risk assessment of clinically heterogeneous AD. First, we took a traditional approach and developed and assessed the utility of a multi-marker genetic risk score to predict AD. The risk score was based on a Discovery cohort association study that sought to replicate previous AD findings and assess additional candidate variants for their association with disease risk. The risk score was then tested for its predictive ability in a separate Validation cohort. Second, we used a more novel decision tree analysis [22] to identify genetic and demographic risk factors for AD. This data-driven method has been used in diverse clinical contexts [23-26] to predict binary outcomes, but is largely unutilized in the prediction of AD diagnosis. It allowed us to assess step-wise interactions between variables to identify the factors that best predict AD.

## Methods

### Participants

Individuals 65- to 101-years-old (N = 216 males, N = 232 females) were evaluated at the University of California, San Francisco Memory and Aging Center (UCSF MAC) and had genotype data available for analysis. All participants were unrelated Caucasians (confirmed by multi-dimensional scaling (MDS) plots or self-described for those without GWAS data available). Non-Caucasians were excluded due to the insufficient number of participants and potential for confounding background genetics. All aspects of the study were approved by the UCSF Institutional Review Board and written informed consent was obtained from all participants and surrogates (as per UCSF Institutional Review Board protocol).

### Clinical assessment

All participants underwent a multi-step screening process with an in-person visit at the MAC that included a neurologic exam, cognitive assessment [27], and medical history. Each participant’s study partner was also interviewed regarding functional abilities. A multidisciplinary team composed of a neurologist, neuropsychologist, and nurse then reviewed all potential participants. Participants included in this study had a study partner (*i.e.*, spouse, close friend). The multidisciplinary team established clinical diagnoses for cases according to consensus criteria for AD [16]. Atypical or concomitant diagnoses were established for lvPPA [16,18], PCA syndrome [16,17], primary executive AD [16], vascular disease [28], or dementia with Lewy bodies (DLB) [29] according to consensus criteria. Individuals with primarily amnestic AD presentations were considered “AmnAD” and those with less common clinical syndromes (lvPPA, PCA, primary executive) or comorbidities (vascular disease, DLB) were considered as “AtAD”. All control subjects underwent a similar multi-step screening process, including study partner interview and a consensus team of clinicians then reviewed all potential participants. Controls included in this study had Mini-Mental State Exam (MMSE) [30] scores ≥26 or a Clinical Dementia Rating Scale (CDR) [31] of 0, no participant or informant report of cognitive decline in the prior year, and no evidence from their screening visit suggesting a neurodegenerative disorder (per team neurologist’s clinical judgment). Individuals harboring a known disease mutation were excluded from the study.

### Genotypes

Genomic DNA was extracted from peripheral blood using standard protocols (Gentra PureGene Blood Kit, QIAGEN, Inc. – USA, Valencia, CA). Genotyping was performed using one of three platforms: TaqMan, Sequenom, or via array genotyping. The method used for each variant is provided in the Supplement (Additional file 1). TaqMan Allelic Discrimination Assay was used for *APOE* genotyping (rs429358 and rs7412) and others as noted, and was conducted on an ABI 7900HT Fast Real-Time PCR system (Applied Biosystems, Foster City, CA) according to manufacturer's instructions. Sequenom iPLEX Technology (Sequenom, San Diego, CA) was also used for genotyping a subset of variants as per manufacturer’s instructions. The SpectroAquire and MassARRAY Typer Software packages (Sequenom, San Diego, CA) were used for interpretation and Typer analyzer (v3.4.0.18) was used to review and analyze data. Only genotypes with “Conservative” or “Moderate” quality calls were included in analysis. A subset of genotypes was also obtained from the Illumina Omni1-Quad array genotyping platform (Illumina Inc., San Diego, CA), processed using manufacturer’s instructions.

A total of 75 variants were genotyped in all subjects and analyzed for association with AD risk. These variants are a culmination of different, on-going studies to evaluate the effect of genes involved in neurodegenerative disease, neurodevelopment, social function, behavior, neuropsychiatry, and language on diseases like AD and frontotemporal dementia (FTD). These included polymorphisms previously associated with: 1) risk for AD or other neurodegenerative disease; 2) neuropsychiatric phenotypes implicated in dementia risk (e.g., depression [32-34], dyslexia [27]; 3) cognitive protection [35]. A full list of variants, associated phenotypes, and accompanying references is provided in Additional file 1. Inclusion criteria for analyzed markers were: >80% non-missing genotypes, ≥0.01 minor allele frequency (MAF), and Hardy-Weinberg equilibrium (HWE) P > 0.001. The average call rate was 98% for all variants.

### Analysis

#### Association study

The study cohort was divided into two groups, a first stage “Discovery” cohort for development of the AD risk score and a second stage “Validation” cohort with which to test the risk scoring method developed in the Discovery cohort. We first conducted association analysis of all markers meeting inclusion criteria in the Discovery cohort. Analyses were performed in PLINK as a logistic regression under an additive model [36].

#### Risk scoring

For scoring, we ranked all findings by p-value and then removed SNPs that were in linkage disequilibrium (LD, r^2^ > 0.8) in our dataset; the single most strongly associated SNP of a set of linked markers was retained. Using the unlinked markers we created raw scoring files for each top finding, iteratively adding the next most significant finding to each scoring set (i.e., 1^st^ marker in first set, 1^st^ and 2^nd^ markers in second set, etc.). Reference alleles were established in the scoring files such that all effects were in the same direction of conferring risk (e.g., a SNP with an empirical OR 0.1 for the reference minor allele would be switched such that the major allele was the reference allele for scoring). Using this paradigm, we created scoring sets for the top findings that were not in LD.

We implemented the ‘SNP scoring’ algorithm in PLINK to first assess the predictive ability of each score set (A-Z) in the Discovery dataset for evaluative purposes. We compared the risk scores for each set against the true phenotypes using receiver operating characteristic (ROC) curves and used the resulting area under the curve (AUC) values to determine the optimal score set, with higher AUC values representing better sensitivity and specificity. The optimal score set was determined as follows. First, score sets were evaluated in two ways: 1) by simple consecutive comparisons of AUC values to identify the set at which AUC is largest, and 2) by statistical comparisons of a given set’s ROC curve AUC (AUC_i_) versus the previous set’s ROC curve AUC (AUC_i-1_) and versus the *APOE*-only score’s AUC (AUC_A_). We then iteratively evaluated sets to determine the maximum AUC, stopping when two consecutive sets each resulted in decreases of AUC as compared to the previous set (i.e., AUC_i_ > AUC_i+1_ & AUC_i_ > AUC_i+2_). After determining this optimal set, we used the same scoring file to create risk scores for the Validation cohort and assessed the AUC of the resulting ROC curve to determine the generalization of our risk scoring method in an independent dataset. All ROC analyses were performed in Stata10/MP (StataCorp LP, College Station, TX).

#### Decision tree analysis

To explore and evaluate the diagnostic potential of the genetic variants available with ROC curves, we used the ROC4 software platform (ROC4.22.exe; http://www.stanford.edu/~yesavage/ROC.html). The software utilizes a user-set weight of sensitivity and specificity (kappa) to choose the predictive variable and value that best divides the sample. The sample is then divided on the value of the variable, which is most predictive based on this sensitivity and specificity. Following this, the program performs the same analysis amongst the subgroups created by the previous step. The process continues until a stopping rule is enforced. The output after stopping rules come into place is a “decision tree” which shows the variables and interactions between them in predicting the outcome of interest. We chose a kappa weight of 0.5 in order to balance efficiency (sensitivity and specificity were equally weighted). There were three stopping rules: when subgroup totals were less than 10, when a significance value corresponding to a multiple-testing-corrected Χ^2^ test greater than P = 0.01 was reached, or when a three way interaction was reached. We performed three ROC analyses: one combined analysis of controls and all types of AD patients, one for the controls and AmnAD, and one for controls and AtAD. The ‘gold standard’ binary score was case/control outcome for any AD clinical diagnosis. Additional predictors included sex (0/1 for male/female), age (in years), and all genetic variants passing quality control (0/1/2 for dose of minor frequency allele).

## Results

**Figure 1:**
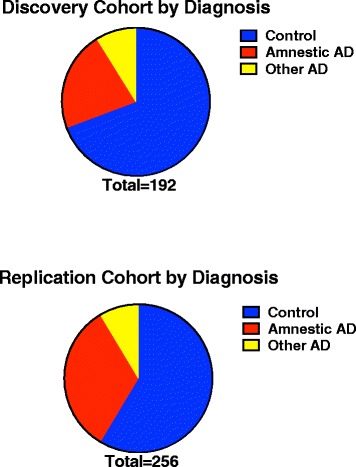
**Diagnosis breakdown.** Each cohort’s composition by diagnosis is shown with controls in blue, amnestic AD in red and other (atypical) AD in yellow.

In total, N = 185 AD cases and N = 283 cognitively normal controls were included in the analysis. Demographics for each group are shown in Table 1. A total of 192 (59 cases, 133 controls) individuals were in the first stage Discovery cohort and 276 (126 cases, 150 controls) were in the second stage Validation cohort. Of the Discovery cohort, 21.9% were AmnAD and 8.9% were AtAD (17 Total, 7 lvPPA, 3 PCA, 3 primarily executive AD, 2 AD with concomitant vascular disease, 2 AD with concomitant DLB; Figure 1). In the Validation cohort, 30.4% were AmnAD, and 8.0% were AtAD (22 Total, 7 lvPPA, 1 PCA, 13 AD with vascular disease, 1 AD with DLB).

**Table 1:** **Sample demographics**

**Discovery**	**AmnAD**	**AtAD**	**Control**
**N**	42	17	133
**Age (mean ± SD)**	76.2 ± 7.9	73.3 ± 6.5	73.9 ± 6.2
**% Female**	42.9%	41.2%	57.1%
**Education (years, mean ± SD)**	16.3 ± 2.5	16.5 ± 2.9	17.3 ± 2.1
**%** ***APOE4*** **carrier**	64.3%	41.2%	20.3%
**Validation**	**AmnAD**	**AtAD**	**Control**
**N**	84	22	150
**Age (mean ± SD)**	80.7 ± 8.4	80.9 ± 9.3	76.8 ± 7.4
**% Female**	53.6%	27.3%	53.3%
**Education (years, mean ± SD)**	15.8 ± 3.1	16.1 ± 4.8	17.4 ± 2.1
**%** ***APOE4*** **carrier**	49.4%	36.4%	24.0%
**Global**	**AmnAD**	**AtAD**	**Control**
**N**	126	39	283
**Age (mean ± SD)**	79.2 ± 8.5	77.6 ± 9.0	75.4 ± 7.0
**% Female**	50.0%	33.3%	55.1%
**Education (years, mean ± SD)**	16.0 ± 2.9	16.7 ± 3.1	17.4 ± 2.1
**%** ***APOE4*** **carrier**	55.2%	38.5%	22.3%

Demographic summary of amnestic Alzheimer’s disease (AmnAD) cases, atypical AD (AtAD) cases and controls.

### Confirmation of AD risk variants and establishment of a 17-marker risk assessment

We first performed an association study in the Discovery cohort as a small-scale replication study of previously identified risk variants for AD in our clinically heterogeneous cohort. We then used this analysis to establish a ranked order by which we could iteratively add variants into a polygenic score to evaluate their utility for risk assessment. In our analysis, only the well-established *APOE* ε4 allele (P = 1.36 × 10^−6^), with an estimated OR = 4.28, met strict significance after Bonferroni correction for multiple testing (Table 2). Seven other variants had nominal p-values of P < 0.05. The second strongest association was with the rs1799945 SNP in *HFE* (P = 1.64 × 10^−3^, OR = 2.83). Variation in the hemochromatosis gene has previously been associated with AD in numerous large meta-analyses [37-39]. Two established risk factors for AD identified by GWAS were nominally associated in our study but with an opposite direction of association, rs3851179 in *PICALM* (P = 2.37 × 10^−3^, OR = 1.87) [40,41] and rs6701713 in *CR1* (P = 0.01, OR = 0.42) [40,42]. More novel AD risk candidates implicated by our study included rs2020942 (P = 0.01, OR = 1.81), a SNP tagging the variable number tandem repeat in the serotonin transporter gene, *SLC6A4,* most often associated with depression [43,44]; rs1799913 (P = 0.04, OR = 0.64) in *TPH1*, an established depression risk factor [45] that was recently associated with depression in AD [34]; rs4504469 (P = 0.04, OR = 0.60) in *KIAA0319*, which was associated with dyslexia [46]; and rs1320490 (P = 0.05, OR = 1.63) in *CDC42BPA*, previously associated with reading ability [47].

**Table 2:** **Association results**

**Gene**	**SNP**	**OR**	**STAT**	**P**	**MAF**
*APOE*	rs429358/rs7412	4.28	4.83	1.36E-06	0.20
*HFE*	rs1799945	2.83	3.15	1.64E-03	0.15
*PICALM*	rs3851179	0.47	−3.04	2.37E-03	0.42
*CR1*	rs6701713	0.42	−2.65	0.01	0.19
*SLC6A4*	rs2020942	1.81	2.63	0.01	0.40
*TPH1*	rs1799913	0.64	−2.02	0.04	0.44
*KIAA0319*	rs4504469	0.60	−2.02	0.04	0.35
*CDC42BPA*	rs1320490	1.63	1.93	0.05	0.20
*TMEM175*	rs6599389	0.41	−1.81	0.07	0.08
*SORL1*	rs2070045	1.63	1.74	0.08	0.22
*CNTNAP2*	rs17236239	0.66	−1.61	0.11	0.16
*ATP2C2*	rs8053211	1.45	1.56	0.12	0.43
*CD2AP*	rs9349407	1.48	1.55	0.12	0.29
*TPD52*	rs7814569	1.79	1.53	0.13	0.09
*COMT*	rs4680	0.72	−1.41	0.16	0.50
*C9ORF72*	rs3849942	1.42	1.38	0.17	0.24
*CPE*	rs11186856	0.33	−1.37	0.17	0.33
*SORL1*	rs12285364	0.42	−1.34	0.18	0.04
*RIT2*	rs4130047	0.70	−1.33	0.18	0.31
*MOBP*	rs1768208	0.72	−1.3	0.19	0.29

Top 20 association results in the Discovery cohort. Only *APOE* was significant after Bonferroni correction for multiple testing. *OR* – odds ratio; *STAT* – test statistic; *MAF* – minor allele frequency.

**Figure 2:**
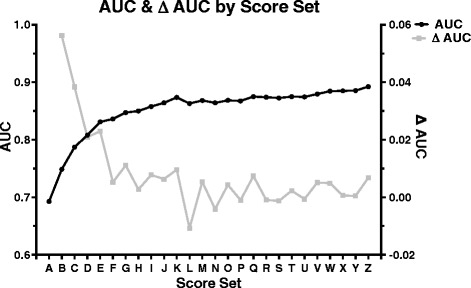
**Area under the curve values for each scoring set.** Area under the curve (AUC) values are provided for each scoring method (in black) as well as the difference between the current and previous scoring method (Δ AUC, in gray). Score sets were iteratively assessed until two consecutive resulting AUC values were lower than the preceding AUC value. The resulting scoring method [“Q”] had an AUC of 0.88.

**Figure 3:**
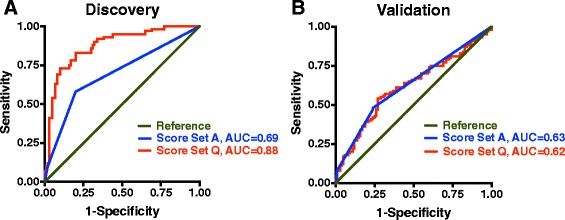
**Receiver operating characteristic curves for Discovery and Validation cohorts.** Receiver operating characteristic (ROC) curves are shown for scoring with *APOE* only (in blue) and the 17-marker risk score (“Q”, in red). AUC values of each curve are provided. **(A)** Discovery cohort shows a significant improvement in sensitivity/specificity as compared to *APOE* only, P < 0.00001. **(B)** Risk scoring with the 17-marker risk score in the Validation cohort does not increase prediction beyond *APOE* genotype alone.

By iteratively adding genetic variants, we found that a risk score panel comprising 17 variants (“Q”) was the best predictor of AD status (Table 3; Figure 2). When evaluated alone, *APOE* genotype had modest predictive value for differentiating AD cases from controls. The 17-marker risk score had a significantly better AUC and was better at predicting AD risk than *APOE* alone (P < 0.00001; Figure 3).

**Table 3:** **Score set evaluation statistics**

**Score**	**AUC ± SE**	**P-val vs. A**	**P-val vs. Prev.**	**ΔAUC**
A	0.69 ± 0.04	N/A	N/A	N/A
B	0.75 ± 0.04	0.01	0.01	0.056
C	0.79 ± 0.03	0.0018	0.02	0.038
D	0.81 ± 0.03	0.00010	0.18	0.021
E	0.83 ± 0.03	<0.00001	0.13	0.023
F	0.84 ± 0.03	<0.00001	0.64	0.005
G	0.85 ± 0.03	<0.00001	0.36	0.011
H	0.85 ± 0.03	<0.00001	0.76	0.003
I	0.86 ± 0.03	<0.00001	0.35	0.008
J	0.86 ± 0.03	<0.00001	0.42	0.006
K	0.87 ± 0.03	<0.00001	0.27	0.010
L	0.86 ± 0.03	<0.00001	0.11	−0.011
M	0.87 ± 0.03	<0.00001	0.35	0.005
N	0.86 ± 0.03	<0.00001	0.55	−0.004
O	0.87 ± 0.03	<0.00001	0.57	0.004
P	0.87 ± 0.03	<0.00001	0.76	−0.001
**Q**	**0.88 ± 0.03**	**<0.00001**	**0.02**	**0.007**
R	0.87 ± 0.03	<0.00001	0.90	−0.001
S	0.87 ± 0.03	<0.00001	0.82	−0.001
T	0.88 ± 0.03	<0.00001	0.67	0.002
U	0.87 ± 0.03	<0.00001	0.88	−0.001
V	0.88 ± 0.03	<0.00001	0.30	0.005
W	0.88 ± 0.03	<0.00001	0.27	0.005
X	0.89 ± 0.03	<0.00001	0.89	0.001
Y	0.89 ± 0.03	<0.00001	0.90	0.001
Z	0.89 ± 0.02	<0.00001	0.25	0.007

Each score set was evaluated for AUC of the ROC curve to assess predictive ability. Score Q (in bold) was determined the best performing scoring set given the following two sets resulted in consecutively lower AUC values. This resulted in a final score set, Q that had a statistically significant better AUC than just *APOE* (set A) alone, P < 0.00001.

### Genetic risk score does not predict AD better than *APOE* in a separate cohort

**Figure 4:**
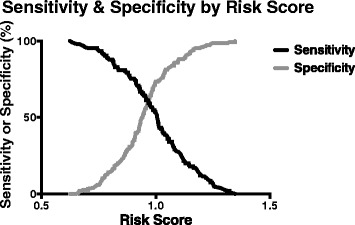
**Sensitivity and specificity for 17-marker scoring method in Validation cohort.** Percent sensitivity (black) and specificity (gray) are plotted by numeric value based on the 17-marker scoring method. Accepting sensitivity of 80% would render specificity of only 36%; specificity of 80% would reduce sensitivity to 45.5%.

When evaluated in the Validation cohort, the “Q” risk scoring method did not perform better than *APOE* alone (Table 4; Figure 3). The 17-marker gene score resulted in 65% maximal correct classification of individuals, with a limited sensitivity (54%) and specificity (73%; Figure 4). Removing excess AmnAD patients from the Validation group to better match the proportion of AtAD individuals in the Discovery cohort did not improve the performance of the multi-marker risk score (Additional file 2).

**Table 4:** **Risk scoring results for the Discovery and Validation cohorts**

	**Score**	**AUC ± SE**	**P-val vs. A**	**N**
**Discovery**	A	0.69 ± 0.04	N/A	192
	Q	0.88 ± 0.03	<0.00001	
**Validation**	A	0.63 ± 0.03	N/A	256
	Q	0.62 ± 0.04	0.7345	

Evaluation metrics for the *APOE*-only risk score (“A”) and the 17-marker risk score (“Q”). The 17-marker risk score predicted AD significantly better than *APOE* alone in the Discovery (P < 0.00001) but not the Validation cohort. Area under the curve (AUC) of the Receiver Operating Characteristic curves for A and Q and p-values of their comparisons are provided.

### Decision tree analysis identifies genetic heterogeneity in amnestic versus atypical AD

We postulated that the clinical heterogeneity between the Discovery and Validation cohorts might be contributing to the failure of the 17-variant risk score to differentiate AD cases from controls better than *APOE* genotype alone. Under an alternative model, the genetic risk for AmnAD is different from that for AtAD. In order to identify genetic and/or demographic criteria that are most useful for accurately differentiating all AD cases from controls and to test whether AmnAD and AtAD share disease predictors or are distinct in their risk profiles, we performed data-driven decision tree analyses. We performed three analyses, one in all AD cases (N = 165) versus controls (N = 283), one with only AmnAD (N = 126) versus controls, and one with AtAD (N = 39) versus controls.

**Figure 5:**
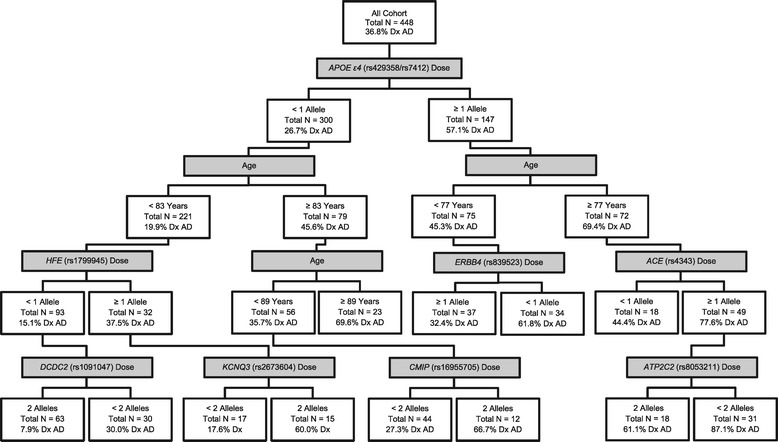
**Decision tree for all forms of Alzheimer’s disease.** Binary decision tree created by receiver operator characteristic (ROC) analysis is shown. Branching points represent the variable and cutting point which best predicts whether or not an individual will be diagnosed with any form of Alzheimer’s disease (AD). Shaded boxes depict the variable used to separate each subgroup and unshaded boxes provide summary data characterizing each subgroup. For more information on the genes depicted, please see Additional file 1.

In the analysis with all AD cases, carrying an *APOE* ε4 allele was the first differentiator of cases from controls (Figure 5). Amongst individuals carrying the ε4 risk allele, the next risk predictor was being ≥77 years old. Of these eldest individuals, the next differentiator was carrying one or more of the minor allele for rs4343 in *ACE,* an AD-risk gene [48,49]. The fourth differentiator of this subgroup was being homozygous for the major allele of rs8053211 in *ATP2C2*, a gene associated with dyslexia and other language traits [50,51], as carriers of one or two copies of the minor allele had a higher risk for diagnosis of AD. Using these predictors, the model had a predictive value positive (PVP) of 0.87, meaning that it correctly predicted a positive AD diagnosis 87% of the time. The sensitivity at this cut point was 0.71 and the specificity was 0.64 (Additional file 3). On the other side of the tree, in individuals carrying no ε4 alleles, the next differentiator of controls from cases was being <83 years old. Of these individuals, not carrying any of the *HFE* SNP, rs1799945, AD risk alleles was more predictive of control status. Finally, carrying two minor alleles of the *DCDC2* SNP rs1091047 (a dyslexia gene [52]) was most predictive of control status. In this final group, the model had a predictive value negative (PVN) of 0.92, meaning it correctly predicted a diagnosis of control 92% of the time. The sensitivity and specificity at this cut point were 0.64 and 0.73, respectively (Additional file 3).

**Figure 6:**
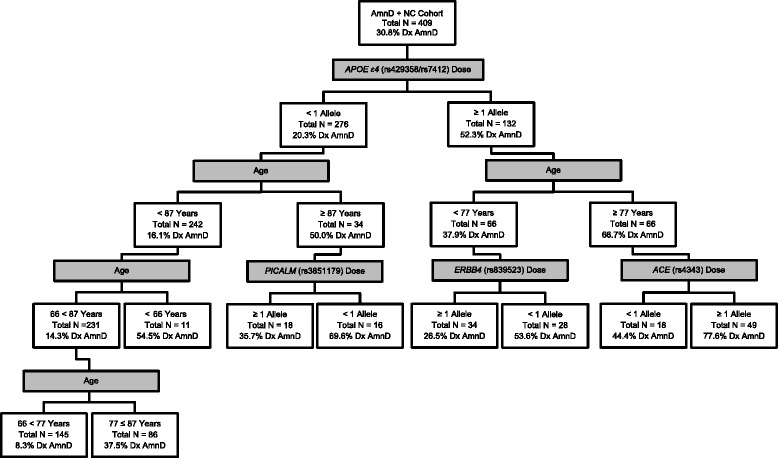
**Decision tree for amnestic Alzheimer’s disease.** Binary decision tree created by receiver operator characteristic (ROC) analysis is shown. Branching points represent the variable and cutting point which best predicts whether or not an individual will be diagnosed with an amnestic form of Alzheimer’s disease (AmnD). Shaded boxes depict the variable used to separate each subgroup and unshaded boxes provide summary data characterizing each subgroup. For more information on the genes depicted, please see Additional file 1. NC - normal control.

In the analysis of AmnAD cases versus controls, carrying an *APOE* ε4 allele was also the best differentiator of cases from controls (Figure 6). Similar to the all-AD analysis, in individuals carrying the ε4 risk allele, the next risk predictor was being ≥77 years old. Of these eldest individuals, the third differentiator was carrying one or more of the minor allele for rs4343 in *ACE.* In these individuals at this cut point, the PVP was 0.76. The sensitivity at this cut point was 0.83 and the specificity was 0.48. On the other side of the tree, in individuals carrying no ε4 alleles, the next differentiator of controls from cases was being between 66–87 years old. In these older individuals, there was another age differentiation whereby being 66–77 years old predicted control status. In this final group, the PVN was 0.92. The sensitivity and specificity at this cut point were 0.64 and 0.67, respectively.

**Figure 7:**
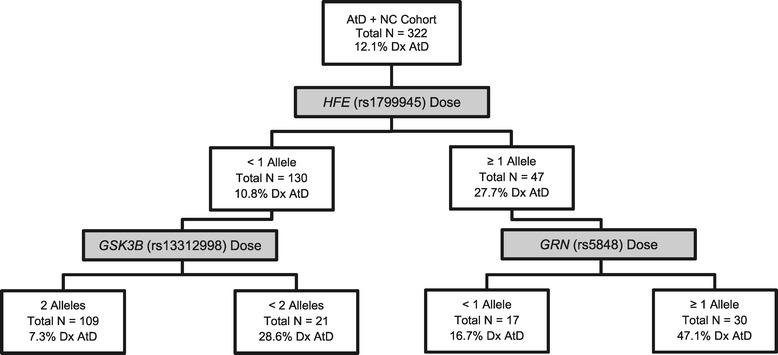
**Decision tree for atypical Alzheimer’s disease.** Binary decision tree created by receiver operator characteristic (ROC) analysis is shown. Branching points represent the variable and cutting point which best predicts whether or not an individual will be diagnosed with an atypical form of Alzheimer’s disease (AtD). Shaded boxes depict the variable used to separate each subgroup and unshaded boxes provide summary data characterizing each subgroup. For more information on the genes depicted, please see Additional file 1. NC - normal control.

The analysis of AtAD cases versus controls provided striking contrast to the previous analyses. In this cohort, carrying one or more minor alleles of the *HFE* SNP (rs1799945) was the first differentiator (Figure 7). In those with *HFE* risk alleles, the next differentiator was carrying ≥1 allele of the *GRN* variant, rs5848, which has been associated with risk for AD [53], hippocampal sclerosis [54,55], FTD [56], and bipolar disorder [57]. In the final at-risk group, the PVP was 0.47, with sensitivity and specificity of 0.62 and 0.74, respectively. On the other side of the tree, the next differentiator predicting control status was being homozygous for the minor allele of *GSK3B* SNP rs13312998, which has also been associated with AD and FTD [58]. At this cut point, the PVN was 0.93. The sensitivity and specificity were 0.43 and 0.87, respectively.

## Discussion

In our association study, we found continuing support for *APOE, HFE, PICALM, CR1, SLC6A4, CDC42BP, TPH1,* and *KIAA0319* as genetic risk factors for AD. Using information from 17 variants combined into a genetic risk score allowed us to predict clinically heterogeneous AD cases significantly better than *APOE* genotype alone, supporting the role of these variants as predictors of AD risk in this primary Discovery group. However, when we attempted to apply this polygenic risk assessment to an independent cohort of clinically heterogeneous AD patients for validation, the utility of analyzing 17 variants was not significantly better than analyzing *APOE* alone. Taken together, this suggests two things. First, it suggests that *APOE* ε4 remains the best predictor of AD risk, likely due to its strong effect, when compared to multiple other risk factors with very modest risk effects. Second, it suggests that phenotypic variability in AD complicates simple genetic risk modeling, particularly when co-morbidities are suspected.

The fact that *APOE* ε4 is the most predictive variant for amnestic AD but does not appear to be associated with risk for atypical AD syndromes such as PCA and lvPPA [59] likely contributes to the decreased specificity of the genetic risk assessment; namely, carrying an ε4 allele is associated with being affected in amnestic AD but is also associated with *not* being affected by PCA or lvPPA. Thus, *APOE* ε4 in the simple context of amnestic AD is quite adept at predicting who will be a case versus control, but is much less specific in the broader context of all AD syndromes, inclusive of atypical presentations and co-morbidities. Indeed, in our entire cohort of Discovery + Validation samples, *APOE* ε4 was significantly enriched in AmnAD but not AtAD cases when compared to controls (AmnAD vs Control P = 3.08 × 10^−7^; AtAD vs Control P = 0.1). A similar discrepancy due to clinical heterogeneity may also underlie our association of variants in *PICALM* and *CR1* in the opposite direction of historical findings. An alternate methodology to identify genetic and demographic factors that predict case/control status in AmnAD and AtAD separately was able to improve differentiation. Utilizing a decision tree methodology, we found that *APOE* best differentiated cases from controls only in AmnAD but not AtAD. In contrast, *HFE* genotype was the best differentiating factor between AtAD cases and controls; the same variant was also the first genetic risk factor for broad AD in individuals without *APOE* ε4. These findings are consistent with prior research implicating *HFE* in AD risk in individuals without *APOE* ε4 [60]. These results also suggest that atypical presentations could represent a distinct genetic class of AD, although the present study was not designed to specifically address this question. A recent study suggests that AtAD is more heritable than AmnAD [61], supporting the theory that there are additional genetic risk factors for AtAD that remain to be elucidated. In the future, GWAS of larger, more diverse cohorts of individuals with specific atypical phenotypes (e.g., PCA) could identify novel genetic risk factors specific to these AD syndromes. Phenotypic specificity in studies of amnestic AD may also provide additional statistical power to identify risk factors of small effect size.

In an effort to rule out the possibility of misdiagnosis, particularly in the AtAD group, we performed a *post hoc* chart review of patients for which pathological data was available (N = 25 AmnAD and N = 8 AtAD). All of these individuals had AD pathology cited as a primary (N = 24 AmnAD, N = 5 AtAD) or major contributing factor (N = 1 AmnAD, N = 3 AtAD) that correlated with each patient’s clinical presentation (Additional file 4). Although not exhaustive, this data suggests that AD pathology was correctly recognized as a major contributor to patients’ clinical syndrome in our patient cohort, and that the differential genetic risk profile of AtAD potentially influences its pathological heterogeneity when compared to AmnAD.

This study benefits from a two-staged discovery-validation study design, inclusion of a broad spectrum of clinical patients representing the phenotypic heterogeneity of AD, well-characterized cognitively normal controls, and inclusion of many of the most replicated genetic loci implicated in AD as well as several, more novel gene candidates. The main limitations of this study include the limited sample size, lack of pathological confirmation in all study participants, and the relatively young age of the controls. In addition, Caucasian individuals were the sole participants in our study, which potentially limits the scope of our findings. Co-morbid depression was not assessed in this analysis and may be a contributing factor to the associations with the depression associated variants. This hypothesis requires direct testing in a separate study.

We implemented a decision tree analysis to identify genetic and demographic criteria most useful for accurately differentiating AD cases from controls. With an iterative, non-parametric approach, we used recursive partitioning to identify individuals according to a binary outcome of interest [22]. This method benefits from limiting the use of restrictive assumptions like linearity, additivity, and homoscedasticity, which are required by most linear models [23]. This approach has been used in a variety of clinical settings to identify variables of interest in predicting binary outcomes such as identification of AD patients who will have rapid cognitive decline [24], presence of tuberculosis after multiple conflicting tests [25], and ability to succeed in diabetes self-management programs [26]. Decision trees are amenable for use in a clinical setting, where an individual’s risk for the outcome of interest—in this case, AD—can be estimated based on multiple predictive variables that follow a logical progression. Testing whether the factors identified in our decision tree analyses have predictive value in a larger, independent cohort will be critical for elucidating whether this risk assessment has clinical utility, particularly with the inclusion of pathologically confirmed cases and exclusion of amyloid-positive ‘controls.’

## Conclusions

We found that *APOE* genotype is the best predictor of risk compared to a polygenic risk score when assessing groups of clinically heterogeneous AD patients versus healthy older controls. In decision tree analysis, we found that AmnAD and AtAD have differential genetic risk factors, which may account for the inaccuracy of the traditional polygenic scoring method. Identifying individuals at highest genetic risk for AD could potentially allow for earlier diagnosis and intervention, allowing the opportunity to intervene with pathological processes and/or provide support prior to clinical onset of symptoms. These risk assessments will benefit from future work to characterize genetic risk factors of clinically homogeneous subtypes of AD in large, diverse populations.

## Additional files

Additional file 1:
**Variants and Genotyping Methods.**
Additional file 2:
**Results for Three Different Versions of the Validation Cohort.**
Additional file 3:
**Decision Tree Analysis Results.**
Additional file 4:
**Pathological Diagnoses for Selected Participants.**
