# Supplementary material for: Decision tree analysis of genetic risk for clinically heterogeneous Alzheimer’s disease
Source: BMC Neurol. 2015 Mar 28;15:47. doi: 10.1186/s12883-015-0304-6 (PMC4459447; doi:10.1186/s12883-015-0304-6)
Supplement: Additional file 1: — Variants and Genotyping Methods. [file 12883_2015_304_MOESM1_ESM.docx]

**Additional File 1: Variants and Genotyping Methods**

|  |  |  |  |  |  |  | | |  |
| --- | --- | --- | --- | --- | --- | --- | --- | --- | --- |
|  | **Gene Name** | **Identifier** | **Reference Allele (A1)** | **Trait/Disease** | **Method** | **Reference** |  |  |  |
|  | *ACE* | rs4343 | A | AD | Sequenom | (Kehoe et al., 1999) [1] |  |  |  |
|  | *APOE* | rs429358/rs7412 | ε4 | AD | Taqman Panel | (Roses et al., 1996) [2] |  |  |  |
|  | *ATP2C2* | rs8053211 | G | ADHD | Sequenom | (Lesch et al., 2008) [3] |  |  |  |
|  | *ATP2C2* | rs11860694 | C | ADHD | Sequenom | (Lesch et al., 2008) [3] |  |  |  |
|  | *BDNF* | rs6265 | A | AD | Sequenom | (Huang et al., 2007) [4] |  |  |  |
|  | *C2ORF3* | rs917235 | G | Dyslexia | Sequenom | (Scerri et al., 2012) [5] |  |  |  |
|  | *C9ORF72* | rs10757668 | T | FTD-ALS | Sequenom | (De Jesus-Hernandez et al., 2011) [6] |  |  |  |
|  | *C9ORF72* | rs3849942 | T | FTD-ALS | Taqman Panel | (Shatunov et al., 2010) [7]; (Dobson-Stone et al., 2012) [8] |  |  |  |
|  | *CD2AP* | rs9349407 | C | AD | Sequenom | (Naj et al., 2011) [9] |  |  |  |
|  | *CDC42BPA* | rs1320490 | C | Myotonic Dystrophy | Sequenom | (Leung et al., 1998) [10] |  |  |  |
|  | *CETP* | rs5882 | G | Memory, Dementia | Sequenom | (Sanders et al., 2010 [11]); (Barzilai et al., 2006) [12] |  |  |  |
|  | *CLU* | rs11136000 | T | AD | Sequenom | (Harold et al., 2009 [13]); (Lambert et al., 2009) [14] |  |  |  |
|  | *CMIP* | rs6564903 | T | Dyslexia, SLI | Sequenom | (Scerri et al., 2011) [15] |  |  |  |
|  | *CMIP* | rs16955705 | C | Dyslexia, SLI | Sequenom | (Scerri et al., 2011) [15] |  |  |  |
|  | *CNTNAP2* | rs4431523 | C | Autism | Sequenom | (Arking et al., 2008) [16] |  |  |  |
|  | *COMT* | rs4680 | A | Psychosis | Sequenom | (Sweet et al., 2005) [17] |  |  |  |
|  | *CR1* | rs6701713 | A | AD | Sequenom | (Lambert et al., 2009) [14] |  |  |  |
|  | *CTNNA2* | rs1007371 | T | Schizophrenia, Handedness | Sequenom | (Francks et al., 2007) [18] |  |  |  |
|  | *CTNNA2* | rs1446109 | G | Schizophrenia, Handedness | Sequenom | (Francks et al., 2007) [18] |  |  |  |
|  | *CTNNA2* | rs723524 | T | Schizophrenia, Handedness | Sequenom | (Francks et al., 2007) [18] |  |  |  |
|  | *CUGBP2* | rs201119 | C | AD | Sequenom | (Wijsman et al., 2011) [19] |  |  |  |
|  | *CYP46A1* | rs754203 | C | AD | Sequenom | (Kolsch et al., 2008) [20] |  |  |  |
|  | *DCDC2* | rs1091047 | C | Dyslexia | Sequenom | (Scerri et al., 2011) [15] |  |  |  |
|  | *DCDC2* | rs793862 | A | Dyslexia | Sequenom | (Scerri et al., 2011) [15] |  |  |  |
|  | *DCDC2* | rs1419228 | G | Dyslexia | Sequenom | (Scerri et al., 2011) [15] |  |  |  |
|  | *DPF3* | rs2192595 | A | Dyslexia | Sequenom | (Meaburn et al., 2008) [21] |  |  |  |
|  | *DRD2* | rs1800497 | T | Creativity | Taqman Panel | (Reuter et al., 2006) [22] |  |  |  |
|  | *DYX1* | rs17819126 | T | Dyslexia | Sequenom | (Nothen et al., 1999) [23] |  |  |  |
|  | *DYX1* | rs57809907 | A | Dyslexia | Sequenom | (Nothen et al., 1999) [23] |  |  |  |
|  | *EIF2AK3* | rs7571971 | T | PSP | Sequenom | (Hoglinger et al., 2011) [24] |  |  |  |
|  | *ERBB4* | rs839523 | A | Schizophrenia, Connectivity | Sequenom | (Law et al., 2007) [25]; (Konrad et al., 2009) [26] |  |  |  |
|  | *EXT2* | chr11:44149719 | T | Autism | Sequenom | (Li et al., 2002) [27] |  |  |  |
|  | *FAM47E* | rs6812193 | T | PD | Sequenom | (Simon-Sanchez et al., 2009) [28] |  |  |  |
|  | *FOXP2* | rs17137124 | T | Dyslexia | Sequenom | (Nudel & Newbury , 2013) [29] |  |  |  |
|  | *GRIN2A* | rs4998386 | T | PD | Sequenom | (Hamza et al., 2011) [30] |  |  |  |
|  | *GRN* | chr17:39784064 | A | FTD | Sequenom | (Caso et al., 2014) [31] |  |  |  |
|  | *GRN* | rs5848 | A | FTD | Sequenom | (Caso et al., 2014) [31] |  |  |  |
|  | *GSK3B* | rs13312998 | A | AD, FTD | Sequenom | (Schaffer et al., 2008) [32] |  |  |  |
|  | *HFE* | rs1799945 | G | Dementia | SNP Array | (Percy et al., 2014) [33] |  |  |  |
|  | Intergenic (Between *QKI* & *LOC7283136*) | rs4145454 | T | AD | Sequenom | (Wijsman et al., 2011) [19] |  |  |  |
|  | *KCNQ3* | rs2673604 | G | AD | Sequenom | (Wijsman et al., 2011) [19] |  |  |  |
|  | *KIAA0319* | rs4504469 | T | Dyslexia | Sequenom | (Velayos-Baeza et al., 2007) [34] |  |  |  |
|  | *KIAA0319L* | rs7523017 | A | Dyslexia | Sequenom | (Couto et al., 2008) [35] |  |  |  |
|  | *KLOTHO* | rs9536314 | G | Aging | Sequenom | (Arking et al., 2002) [36] |  |  |  |
|  | *KLOTHO* | rs9527025 | C | Aging | Sequenom | (Arking et al., 2002) [36] |  |  |  |
|  | *KLOTHO* | rs7997728 | G | Aging | Sequenom | (Arking et al., 2002) [36] |  |  |  |
|  | *KLOTHO* | rs9536312 | T | Aging | Sequenom | (Arking et al., 2002) [36] |  |  |  |
|  | *MAP1B* | chr5:71529485 | C | Adult ADHD | Sequenom | (Lesch et al., 2008) [3] |  |  |  |
|  | *MAPT* | rs1560310 | H2 | FTD, AD | Taqman Panel | (Coppola et al., 2012) [37] |  |  |  |
|  | *MCCC1* | rs10513789 | G | PD | Sequenom | (Do et al., 2011) [38] |  |  |  |
|  | *MOBP* | rs1768208 | T | PSP | Sequenom | (Hoglinger et al., 2011) [24] |  |  |  |
|  | *NRG* | rs35753505 | C | Psychosis, Memory | Sequenom | (Krug et al., 2008) [39] |  |  |  |
|  | *OXTR* | rs53576 | A | Social behavior, Empathy | Sequenom | (Bakermans-Kranenburg and van Ijzendoorn, 2008) [40]; (Rodrigues et al., 2009) [41] |  |  |  |
|  | *PARK7* | chr1:7953581 | A | PD | Sequenom | (Le & Appel, 2003) [42] |  |  |  |
|  | *PCDH11X* | rs5984894 | A | AD | Sequenom | (Carrasquillo et al., 2009) [43] |  |  |  |
|  | *PICALM* | rs3851179 | A | AD | Sequenom | (Harold et al., 2009) [13] |  |  |  |
|  | *PSEN2* | chr1:225138072 | A | AD | Sequenom | (Cruchaga et al., 2012) [44] |  |  |  |
|  | *RAI1* | rs11649804 | A | PD | Sequenom | (Do et al., 2011) [15] |  |  |  |
|  | *RIT2* | rs4130047 | C | PD | Sequenom | (Do et al., 2011) [38] |  |  |  |
|  | *SLC41A1* | rs823156 | G | PD, | Sequenom | (Do et al., 2011) [38] |  |  |  |
|  | *SLC6A4* | rs2020942 | A | Depression, Psychosis | Sequenom | (Fan and Sklar, 2005) [45]; (Wray et al., 2009) [46] |  |  |  |
|  | *SNAP25* | rs1051312 | C | Memory, ADHD | Sequenom | (Forero et al., 2009) [47] |  |  |  |
|  | *SNCA* | rs356220 | T | PD | Sequenom | (Do et al., 2011) [38] |  |  |  |
|  | *SNCAIP* | chr5:121814858 | A | PD | Sequenom | (Chung et al., 2001) [48] |  |  |  |
|  | *SORL1* | rs2070045 | G | AD | Sequenom | (Rogaeva et al., 2007) [49] |  |  |  |
|  | *SORL1* | rs12285364 | T | AD | Sequenom | (Rogaeva et al., 2007) [49] |  |  |  |
|  | *SORL1* | rs3824968 | A | AD | Sequenom | (Rogaeva et al., 2007) [49] |  |  |  |
|  | *SORL1* | rs661057 | C | AD | Sequenom | (Rogaeva et al., 2007) [49] |  |  |  |
|  | *SREBP1* | rs11868035 | A | PD | Sequenom | (Do et al., 2011) [38] |  |  |  |
|  | *STX6* | rs1411478 | A | PSP | Sequenom | (Hoglinger et al., 2011) [24] |  |  |  |
|  | *TDP52* | rs7814569 | G | AD | Sequenom | (Wijsman et al., 2011) [19] |  |  |  |
|  | *TMEM106B* | rs1990622 | C | FTD | Sequenom | (Van Deerlin et al., 2010) [37] |  |  |  |
|  | *TMEM175* | rs6599389 | A | PD | Sequenom | (Do et al., 2011) [38] |  |  |  |
|  | *TPH1* | rs1799913 | A | Creativity | Taqman Panel | (Reuter et al., 2006) [22] |  |  |  |
|  | *TTRAP* | rs2143340 | C | Dyslexia | Sequenom | (Francks et al., 2004) [50] |  |  |  |
|  |  |  |  |  |  |  |  |  |  |

**Additional File 1 Table Legend:** Gene name, variant identifier (provided as an rs number except for those variants not in dbSNP, in which case chromosome and base position based on hg19 is given), reference allele for genotyping, associated disease or trait, genotyping method, and citation of association are provided for each marker assessed for association with Alzheimer’s disease risk. AD – Alzheimer’s disease; ADHD – Attention Deficit Hyperactivity Disorder; FTD-ALS – Frontotemporal dementia and amyotrophic lateral sclerosis; SLI – Specific language impairment; PSP – Progressive supranuclear palsy; FTD – Frontotemporal dementia; PD – Parkinson’s disease; BPD – Bipolar Disorder.

**REFERENCES**

1. Kehoe PG, Katzov H, Feuk L, Bennet AM, Johansson B, Wilman B, de Faire U, Cairns NJ, Wilcock GK, Brookes AJ, Blennow K, Prince JA: **Haplotypes extending across ACE are associated with Alzheimer’s disease**. *Hum Mol Genet* 2003, **12**:859–867.

2. Roses AD: **Apolipoprotein E alleles as risk factors in Alzheimer’s disease.** *Annu Rev Med* 1996, **47**:387–400.

3. Lesch KP, Timmesfeld N, Renner TJ, Halperin R, Röser C, Nguyen TT, Craig DW, Romanos J, Heine M, Meyer J, Freitag C, Warnke A, Romanos M, Schäfer H, Walitza S, Reif A, Stephan DA, Jacob C: **Molecular genetics of adult ADHD: Converging evidence from genome-wide association and extended pedigree linkage studies**. *J Neural Transm* 2008, **115**:1573–1585.

4. Huang R, Huang J, Cathcart H, Smith S, Poduslo SE: **Genetic variants in brain-derived neurotrophic factor associated with Alzheimer’s disease.** *J Med Genet* 2007, **44**:e66.

5. Scerri TS, Darki F, Newbury DF, Whitehouse AJO, Peyrard-Janvid M, Matsson H, Ang QW, Pennell CE, Ring S, Stein J, Morris AP, Monaco AP, Kere J, Talcott JB, Klingberg T, Paracchini S: **The dyslexia candidate locus on 2p12 is associated with general cognitive ability and white matter structure.** *PLoS One* 2012, **7**:e50321.

6. DeJesus-Hernandez M, Mackenzie IR, Boeve BF, Boxer AL, Baker M, Rutherford NJ, Nicholson AM, Finch NA, Flynn H, Adamson J, Kouri N, Wojtas A, Sengdy P, Hsiung G-YR, Karydas A, Seeley WW, Josephs K a, Coppola G, Geschwind DH, Wszolek ZK, Feldman H, Knopman DS, Petersen RC, Miller BL, Dickson DW, Boylan KB, Graff-Radford NR, Rademakers R: **Expanded GGGGCC hexanucleotide repeat in noncoding region of C9ORF72 causes chromosome 9p-linked FTD and ALS.** *Neuron* 2011, **72**:245–56.

7. Shatunov A, Mok K, Newhouse S, Weale ME, Smith B, Vance C, Johnson L, Veldink JH, van Es M a, van den Berg LH, Robberecht W, Van Damme P, Hardiman O, Farmer AE, Lewis CM, Butler AW, Abel O, Andersen PM, Fogh I, Silani V, Chiò A, Traynor BJ, Melki J, Meininger V, Landers JE, McGuffin P, Glass JD, Pall H, Leigh PN, Hardy J, et al.: **Chromosome 9p21 in sporadic amyotrophic lateral sclerosis in the UK and seven other countries: a genome-wide association study.** *Lancet Neurol* 2010, **9**:986–94.

8. Dobson-Stone C, Hallupp M, Bartley L, Shepherd CE, Halliday GM, Schofield PR, Hodges JR, Kwok JBJ: **C9ORF72 repeat expansion in clinical and neuropathologic frontotemporal dementia cohorts**. *Neurology* 2012, **79**:995–1001.

9. Naj AC, Jun G, Beecham GW, Wang L-S, Vardarajan BN, Buros J, Gallins PJ, Buxbaum JD, Jarvik GP, Crane PK, Larson EB, Bird TD, Boeve BF, Graff-Radford NR, De Jager PL, Evans D, Schneider JA, Carrasquillo MM, Ertekin-Taner N, Younkin SG, Cruchaga C, Kauwe JSK, Nowotny P, Kramer P, Hardy J, Huentelman MJ, Myers AJ, Barmada MM, Demirci FY, Baldwin CT, et al.: **Common variants at MS4A4/MS4A6E, CD2AP, CD33 and EPHA1 are associated with late-onset Alzheimer’s disease.** *Nat Genet* 2011, **43**:436–41.

10. Leung T, Chen XQ, Tan I, Manser E, Lim L: **Myotonic dystrophy kinase-related Cdc42-binding kinase acts as a Cdc42 effector in promoting cytoskeletal reorganization.** *Mol Cell Biol* 1998, **18**:130–40.

11. Sanders AE, Wang C, Katz M, Derby CA, Barzilai N, Ozelius L, Lipton RB: **Association of a functional polymorphism in the cholesteryl ester transfer protein (CETP) gene with memory decline and incidence of dementia.** *JAMA* 2010, **303**:150–8.

12. Barzilai N, Atzmon G, Derby C a, Bauman JM, Lipton RB: **A genotype of exceptional longevity is associated with preservation of cognitive function.** *Neurology* 2006, **67**:2170–5.

13. Harold D, Abraham R, Hollingworth P, Sims R, Gerrish A, Hamshere ML, Pahwa JS, Moskvina V, Dowzell K, Williams A, Jones N, Thomas C, Stretton A, Morgan AR, Lovestone S, Powell J, Proitsi P, Lupton MK, Brayne C, Rubinsztein DC, Gill M, Lawlor B, Lynch A, Morgan K, Brown KS, Passmore PA, Craig D, McGuinness B, Todd S, Holmes C, et al.: **Genome-wide association study identifies variants at CLU and PICALM associated with Alzheimer’s disease.** *Nat Genet* 2009, **41**:1088–93.

14. Lambert J-C, Heath S, Even G, Campion D, Sleegers K, Hiltunen M, Combarros O, Zelenika D, Bullido MJ, Tavernier B, Letenneur L, Bettens K, Berr C, Pasquier F, Fiévet N, Barberger-Gateau P, Engelborghs S, De Deyn P, Mateo I, Franck A, Helisalmi S, Porcellini E, Hanon O, de Pancorbo MM, Lendon C, Dufouil C, Jaillard C, Leveillard T, Alvarez V, Bosco P, et al.: **Genome-wide association study identifies variants at CLU and CR1 associated with Alzheimer’s disease.** *Nat Genet* 2009, **41**:1094–9.

15. Scerri TS, Morris AP, Buckingham L-L, Newbury DF, Miller LL, Monaco AP, Bishop DVM, Paracchini S: **DCDC2, KIAA0319 and CMIP are associated with reading-related traits.** *Biol Psychiatry* 2011, **70**:237–45.

16. Arking DE, Cutler DJ, Brune CW, Teslovich TM, West K, Ikeda M, Rea A, Guy M, Lin S, Cook EH, Chakravarti A: **A Common Genetic Variant in the Neurexin Superfamily Member CNTNAP2 Increases Familial Risk of Autism**. *Am J Hum Genet* 2008, **82**:160–164.

17. Sweet R a, Devlin B, Pollock BG, Sukonick DL, Kastango KB, Bacanu S, Chowdari K V, DeKosky ST, Ferrell RE: **Catechol-O-methyltransferase haplotypes are associated with psychosis in Alzheimer disease.** *Mol Psychiatry* 2005, **10**:1026–36.

18. Francks C, Maegawa S, Laurén J, Abrahams BS, Velayos-Baeza A, Medland SE, Colella S, Groszer M, McAuley EZ, Caffrey TM, Timmusk T, Pruunsild P, Koppel I, Lind PA, Matsumoto-Itaba N, Nicod J, Xiong L, Joober R, Enard W, Krinsky B, Nanba E, Richardson AJ, Riley BP, Martin NG, Strittmatter SM, Möller H-J, Rujescu D, St Clair D, Muglia P, Roos JL, et al.: **LRRTM1 on chromosome 2p12 is a maternally suppressed gene that is associated paternally with handedness and schizophrenia.** *Mol Psychiatry* 2007, **12**:1129–39.

19. Wijsman EM, Pankratz ND, Choi Y, Rothstein JH, Faber KM, Cheng R, Lee JH, Bird TD, Bennett DA, Diaz-Arrastia R, Goate AM, Farlow M, Ghetti B, Sweet RA, Foroud TM, Mayeux R: **Genome-wide association of familial late-onset Alzheimer’s disease replicates BIN1 and CLU and nominates CUGBP2 in interaction with APOE.** *PLoS Genet* 2011, **7**:e1001308.

20. Kölsch H, Lütjohann D, Jessen F, Popp J, Hentschel F, Kelemen P, Schmitz S, Maier W, Heun R: **CYP46A1 variants influence Alzheimer’s disease risk and brain cholesterol metabolism.** *Eur Psychiatry* 2009, **24**:183–90.

21. Meaburn EL, Harlaar N, Craig IW, Schalkwyk LC, Plomin R: **Quantitative trait locus association scan of early reading disability and ability using pooled DNA and 100K SNP microarrays in a sample of 5760 children.** *Mol Psychiatry* 2008, **13**:729–40.

22. Reuter M, Roth S, Holve K, Hennig J: **Identification of first candidate genes for creativity: a pilot study.** *Brain Res* 2006, **1069**:190–7.

23. Nöthen MM, Schulte-Körne G, Grimm T, Cichon S, Vogt IR, Müller-Myhsok B, Propping P, Remschmidt H: **Genetic linkage analysis with dyslexia: evidence for linkage of spelling disability to chromosome 15.** *Eur Child Adolesc Psychiatry* 1999, **8 Suppl 3**:56–9.

24. Höglinger GU, Melhem NM, Dickson DW, Sleiman PMA, Wang L-S, Klei L, Rademakers R, de Silva R, Litvan I, Riley DE, van Swieten JC, Heutink P, Wszolek ZK, Uitti RJ, Vandrovcova J, Hurtig HI, Gross RG, Maetzler W, Goldwurm S, Tolosa E, Borroni B, Pastor P, Cantwell LB, Han MR, Dillman A, van der Brug MP, Gibbs JR, Cookson MR, Hernandez DG, Singleton AB, et al.: **Identification of common variants influencing risk of the tauopathy progressive supranuclear palsy.** *Nat Genet* 2011, **43**:699–705.

25. Law AJ, Kleinman JE, Weinberger DR, Weickert CS: **Disease-associated intronic variants in the ErbB4 gene are related to altered ErbB4 splice-variant expression in the brain in schizophrenia.** *Hum Mol Genet* 2007, **16**:129–41.

26. Konrad A, Vucurevic G, Musso F, Stoeter P, Dahmen N, Winterer G: **ErbB4 genotype predicts left frontotemporal structural connectivity in human brain.** *Neuropsychopharmacology* 2009, **34**:641–50.

27. Li H, Yamagata T, Mori M, Momoi MY: **Association of autism in two patients with hereditary multiple exostoses caused by novel deletion mutations of EXT1.** *J Hum Genet* 2002, **47**:262–5.

28. Simón-Sánchez J, Schulte C, Bras JM, Sharma M, Gibbs JR, Berg D, Paisan-Ruiz C, Lichtner P, Scholz SW, Hernandez DG, Krüger R, Federoff M, Klein C, Goate A, Perlmutter J, Bonin M, Nalls M a, Illig T, Gieger C, Houlden H, Steffens M, Okun MS, Racette B a, Cookson MR, Foote KD, Fernandez HH, Traynor BJ, Schreiber S, Arepalli S, Zonozi R, et al.: **Genome-wide association study reveals genetic risk underlying Parkinson’s disease.** *Nat Genet* 2009, **41**:1308–12.

29. Nudel R, Newbury DF: **Foxp2.** *Wiley Interdiscip Rev Cogn Sci* 2013, **4**:547–560.

30. Hamza TH, Chen H, Hill-Burns EM, Rhodes SL, Montimurro J, Kay DM, Tenesa A, Kusel VI, Sheehan P, Eaaswarkhanth M, Yearout D, Samii A, Roberts JW, Agarwal P, Bordelon Y, Park Y, Wang L, Gao J, Vance JM, Kendler KS, Bacanu S-A, Scott WK, Ritz B, Nutt J, Factor S a, Zabetian CP, Payami H: **Genome-wide gene-environment study identifies glutamate receptor gene GRIN2A as a Parkinson’s disease modifier gene via interaction with coffee.** *PLoS Genet* 2011, **7**:e1002237.

31. Caso F, Agosta F, Magnani G, Galantucci S, Spinelli EG, Galimberti D, Falini A, Comi G, Filippi M: **Clinical and MRI correlates of disease progression in a case of nonfluent/agrammatic variant of primary progressive aphasia due to progranulin (GRN) Cys157LysfsX97 mutation.** *J Neurol Sci* 2014, 342:167-172.

32. Schaffer BAJ, Bertram L, Miller BL, Mullin K, Weintraub S, Johnson N, Bigio EH, Mesulam M, Wiedau-Pazos M, Jackson GR, Cummings JL, Cantor RM, Levey AI, Tanzi RE, Geschwind DH: **Association of GSK3B with Alzheimer disease and frontotemporal dementia.** *Arch Neurol* 2008, **65**:1368–1374.

33. Percy M, Somerville MJ, Hicks M, Garcia A, Colelli T, Wright E, Kitaygorodsky J, Jiang A, Ho V, Parpia A, Wong MK: **Risk factors for development of dementia in a unique six-year cohort study. I. An exploratory, pilot study of involvement of the E4 allele of apolipoprotein E, mutations of the hemochromatosis-HFE gene, type 2 diabetes, and stroke.** *J Alzheimers Dis* 2014, **38**:907–22.

34. Velayos-Baeza A, Toma C, da Roza S, Paracchini S, Monaco AP: **Alternative splicing in the dyslexia-associated gene KIAA0319.** *Mamm genome* 2007, **18**:627–34.

35. Couto JM, Gomez L, Wigg K, Cate-Carter T, Archibald J, Anderson B, Tannock R, Kerr EN, Lovett MW, Humphries T, Barr CL: **The KIAA0319-like (KIAA0319L) gene on chromosome 1p34 as a candidate for reading disabilities.** *J Neurogenet* 2008, **22**:295–313.

36. Arking DE, Krebsova A, Macek M, Arking A, Mian IS, Fried L, Hamosh A, Dey S, McIntosh I, Dietz HC: **Association of human aging with a functional variant of klotho.** *Proc Natl Acad Sci U S A* 2002, **99**:856–61.

37. Coppola G, Chinnathambi S, Lee JJ, Dombroski BA, Baker MC, Soto-Ortolaza AI, Lee SE, Klein E, Huang AY, Sears R, Lane JR, Karydas AM, Kenet RO, Biernat J, Wang L-S, Cotman CW, Decarli CS, Levey AI, Ringman JM, Mendez MF, Chui HC, Le Ber I, Brice A, Lupton MK, Preza E, Lovestone S, Powell J, Graff-Radford N, Petersen RC, Boeve BF, et al.: **Evidence for a role of the rare p.A152T variant in MAPT in increasing the risk for FTD-spectrum and Alzheimer’s diseases.** *Hum Mol Genet* 2012, **21**:3500–12.

38. Do CB, Tung JY, Dorfman E, Kiefer AK, Drabant EM, Francke U, Mountain JL, Goldman SM, Tanner CM, Langston JW, Wojcicki A, Eriksson N: **Web-based genome-wide association study identifies two novel loci and a substantial genetic component for Parkinson’s disease.** *PLoS Genet* 2011, **7**:e1002141.

39. Krug A, Markov V, Eggermann T, Krach S, Zerres K, Stöcker T, Shah NJ, Schneider F, Nöthen MM, Treutlein J, Rietschel M, Kircher T: **Genetic variation in the schizophrenia-risk gene neuregulin1 correlates with differences in frontal brain activation in a working memory task in healthy individuals.** *Neuroimage* 2008, **42**:1569–76.

40. Bakermans-Kranenburg MJ, van Ijzendoorn MH: **Oxytocin receptor (OXTR) and serotonin transporter (5-HTT) genes associated with observed parenting.** *Soc Cogn Affect Neurosci* 2008, **3**:128–34.

41. Rodrigues SM, Saslow LR, Garcia N, John OP, Keltner D: **Oxytocin receptor genetic variation relates to empathy and stress reactivity in humans.** *Proc Natl Acad Sci U S A* 2009, **106**:21437–41.

42. Le W, Appel SH: **Mutant genes responsible for Parkinson’s disease.** *Curr Opin Pharmacol* 2004, **4**:79–84.

43. Carrasquillo MM, Zou F, Pankratz VS, Wilcox SL, Ma L, Walker LP, Younkin SG, Younkin CS, Younkin LH, Bisceglio GD, Ertekin-Taner N, Crook JE, Dickson DW, Petersen RC, Graff-Radford NR, Younkin SG: **Genetic variation in PCDH11X is associated with susceptibility to late-onset Alzheimer’s disease.** *Nat Genet* 2009, **41**:192–8.

44. Cruchaga C, Haller G, Chakraverty S, Mayo K, Vallania FLM, Mitra RD, Faber K, Williamson J, Bird T, Diaz-Arrastia R, Foroud TM, Boeve BF, Graff-Radford NR, St Jean P, Lawson M, Ehm MG, Mayeux R, Goate AM: **Rare variants in APP, PSEN1 and PSEN2 increase risk for AD in late-onset Alzheimer’s disease families.** *PLoS One* 2012, **7**:e31039.

45. Fan JB, Sklar P: **Meta-analysis reveals association between serotonin transporter gene STin2 VNTR polymorphism and schizophrenia.** *Mol Psychiatry* 2005, **10**:928–38.

46. Wray NR, James MR, Gordon SD, Dumenil T, Ryan L, Coventry WL, Statham DJ, Pergadia ML, Madden PAF, Heath AC, Montgomery GW, Martin NG: **Accurate, large-scale genotyping of 5HTTLPR and flanking single nucleotides polymorphisms in an association study of depression, anxiety, and personality measures.** *Biol Psychiatry* 2009, **66**:468–76.

47. Forero DA, Arboleda GH, Vasquez R, Arboleda H: **Candidate genes involved in neural plasticity and the risk for attention-deficit hyperactivity disorder: a meta-analysis of 8 common variants.** *J Psychiatry Neurosci* 2009, **34**:361–366.

48. Chung KK, Zhang Y, Lim KL, Tanaka Y, Huang H, Gao J, Ross CA, Dawson VL, Dawson TM: **Parkin ubiquitinates the alpha-synuclein-interacting protein, synphilin-1: implications for Lewy-body formation in Parkinson disease.** *Nat Med* 2001, **7**:1144–50.

49. Rogaeva E, Meng Y, Lee JH, Gu Y, Kawarai T, Zou F, Katayama T, Baldwin CT, Cheng R, Hasegawa H, Chen F, Shibata N, Lunetta KL, Pardossi-Piquard R, Bohm C, Wakutani Y, Cupples LA, Cuenco KT, Green RC, Pinessi L, Rainero I, Sorbi S, Bruni A, Duara R, Friedland RP, Inzelberg R, Hampe W, Bujo H, Song Y-Q, Andersen OM, et al.: **The neuronal sortilin-related receptor SORL1 is genetically associated with Alzheimer disease.** *Nat Genet* 2007, **39**:168–77.

50. Francks C, Paracchini S, Smith SD, Richardson AJ, Scerri TS, Cardon LR, Marlow AJ, MacPhie IL, Walter J, Pennington BF, Fisher SE, Olson RK, DeFries JC, Stein JF, Monaco AP: **A 77-kilobase region of chromosome 6p22.2 is associated with dyslexia in families from the United Kingdom and from the United States.** *Am J Hum Genet* 2004, **75**:1046–58.
