# Supplementary material for: Decision tree analysis of genetic risk for clinically heterogeneous Alzheimer’s disease
Source: BMC Neurol. 2015 Mar 28;15:47. doi: 10.1186/s12883-015-0304-6 (PMC4459447; doi:10.1186/s12883-015-0304-6)
Supplement: Additional file 2: — Results for Three Different Versions of the Validation Cohort. [file 12883_2015_304_MOESM2_ESM.docx]

**Additional File 2: Results for Three Different Versions of the Validation Cohort**

|  | **Score** | **AUC ± SE** | **P-val vs. A** | **N** |
| --- | --- | --- | --- | --- |
| Original Validation Cohort | A | 0.63 ± 0.03 | N/A | 256 |
|  | Q | 0.62 ± 0.04 | 0.73 |  |
| Validation Subset 1 | A | 0.63 ± 0.03 | N/A | 228 |
|  | Q | 0.59 ± 0.04 | 0.39 |  |
| Validation Subset 2 | A | 0.61 ± 0.03 | N/A | 228 |
|  | Q | 0.63 ± 0.04 | 0.6 |  |
| Validation Subset 3 | A | 0.63 ± 0.03 | N/A | 228 |
|  | Q | 0.62 ± 0.04 | 0.68 |  |
| Mean Validation Subsets 1-3 | A | 0.62 ± 0.03 | N/A | 228 |
|  | Q | 0.61 ± 0.04 | 0.56 |  |

**Additional File 2 Legend:** Removing excess amnestic Alzheimer’s disease (AmnAD) patients from the Validation group to better match the proportion of amnestic to atypical AD (AtAD) individuals in the Discovery cohort did not improve the performance of the multi-marker risk score. In each group, Validation 1-3, 28 different AmnAD patients were randomly removed from the group and the comparison of Score A to Score Q was reevaluated in the smaller group of 228 total cases and controls. In each of these iterations, Score A had a mean ± SE area under the ROC curve (AUC) = 0.62 ± 0.03 whereas Score Q had a mean ± SE AUC of 0.61 ± 0.04. This was very similar to the original Validation cohort results, suggesting that altering the ratio of AmnAD to AtAD patients in the Validation cohort does not improve the ability to replicate the initial findings from the Discovery group.
