# Supplementary material for: Decision tree analysis of genetic risk for clinically heterogeneous Alzheimer’s disease
Source: BMC Neurol. 2015 Mar 28;15:47. doi: 10.1186/s12883-015-0304-6 (PMC4459447; doi:10.1186/s12883-015-0304-6)
Supplement: Additional file 3: — Decision Tree Analysis Results. [file 12883_2015_304_MOESM3_ESM.docx]

**Additional File 3: Decision Tree Analysis Results**

| **All AD versus Control** | | | | | | | | | |
| --- | --- | --- | --- | --- | --- | --- | --- | --- | --- |
| **Cut Point Criteria** | **Cut Point** | **PVP** | **PVN** | **Sens.** | **Spec.** | **PP n** | **PN n** | **Χ^2^ value** | **P-Value** |
| *APOE* ε*4* (rs429358/rs7412) | ≥ 1 Allele | 0.57 | 0.73 | 0.51 | 0.78 | 147 | 300 | 39.45 | *** |
| Age | ≥ 77 Years | 0.69 | 0.55 | 0.6 | 0.65 | 72 | 75 | 8.72 | ** |
| Age | ≥ 83 Years | 0.46 | 0.8 | 0.45 | 0.8 | 79 | 221 | 19.6 | *** |
| *ACE* (rs4343) | ≥ 1 Allele | 0.78 | 0.56 | 0.83 | 0.48 | 49 | 18 | 6.71 | ** |
| *ERBB4* (rs839523) | < 1 Allele | 0.62 | 0.68 | 0.64 | 0.66 | 34 | 37 | 6.13 | * |
| Age | ≥ 89 Years | 0.7 | 0.64 | 0.44 | 0.84 | 23 | 56 | 7.53 | ** |
| *HFE* (rs1799945) | ≥ 1 Allele | 0.38 | 0.85 | 0.46 | 0.8 | 32 | 93 | 7.28 | ** |
| *ATP2C2* (rs8053211) | < 2 Alleles | 0.87 | 0.39 | 0.71 | 0.64 | 31 | 18 | 4.42 | * |
| CMIP (rs16955705) | ≥ 2 Alleles | 0.67 | 0.73 | 0.4 | 0.89 | 12 | 44 | 6.37 | * |
| *KCNQ3* (rs2673604) | ≥ 2 Alleles | 0.6 | 0.82 | 0.75 | 0.7 | 15 | 17 | 6.1 | * |
| *DCDC2* (rs1091047) | < 2 Alleles | 0.3 | 0.92 | 0.64 | 0.73 | 30 | 63 | 7.74 | ** |
| **Amnestic AD versus Control** | | | | | | | | | |
| **Cut Point Criteria** | **Cut Point** | **PVP** | **PVN** | **Sens.** | **Spec.** | **PP n** | **PN n** | **Χ^2^ value** | **P-Value** |
| *APOE* ε*4* (rs429358/rs7412) | ≥ 1 Allele | 0.52 | 0.8 | 0.55 | 0.78 | 132 | 276 | 42.98 | *** |
| Age | ≥ 77 Years | 0.67 | 0.62 | 0.64 | 0.65 | 66 | 66 | 10.96 | *** |
| Age | ≥ 87 Years | 0.5 | 0.84 | 0.3 | 0.92 | 34 | 242 | 21.16 | *** |
| *ACE* (rs4343) | ≥ 1 Allele | 0.76 | 0.59 | 0.83 | 0.48 | 45 | 17 | 6.51 | * |
| *ERBB4* (rs839523) | < 1 Allele | 0.54 | 0.74 | 0.63 | 0.66 | 28 | 34 | 4.75 | * |
| *PICALM* (rs3851179) | < 1 Allele | 0.75 | 0.72 | 0.71 | 0.76 | 16 | 18 | 7.56 | ** |
| Age | < 66 Years | 0.55 | 0.86 | 0.15 | 0.98 | 11 | 231 | 12.59 | *** |
| Age | ≥ 77 Years | 0.24 | 0.92 | 0.64 | 0.67 | 86 | 145 | 11.49 | *** |
| **Atypical AD versus Control** | | | | | | | | | |
| **Cut Point Criteria** | **Cut Point** | **PVP** | **PVN** | **Sens.** | **Spec.** | **PP n** | **PN n** | **Χ^2^ value** | **P-Value** |
| *HFE* (rs1799945) | ≥ 1 Allele | 0.28 | 0.89 | 0.48 | 0.77 | 47 | 130 | 7.62 | ** |
| *GRN* (rs5848) | ≥ 1 Allele | 0.47 | 0.83 | 0.62 | 0.74 | 17 | 30 | 5.01 | * |
| *GSK3B* (rs13312998) | < 2 Alleles | 0.29 | 0.93 | 0.43 | 0.87 | 21 | 109 | 8.26 | ** |

**Additional File 3 Legend:** Cut point criteria (either a gene or an age), cut point (allele count or years), predictive value positive (PVP), predictive value negative (PVN), sensitivity (Sens.), specificity (Spec.), number predicted positive (PP n), number predicted negative (PN n), Χ^2^ value from chi-square testing, and P-values corrected for multiple testing ( *, **, and *** equate to corrected P-values of 0.05, 0.01, and 0.001, respectively). When a gene was used as a cut point, an rs number specified the variant. PVP is defined as the proportion correctly predicted as positive at each cut point. PVN is defined as the proportion correctly predicted as negative at each cut point. AD – Alzheimer’s disease.
