# Supplementary material for: Decision tree analysis of genetic risk for clinically heterogeneous Alzheimer’s disease
Source: BMC Neurol. 2015 Mar 28;15:47. doi: 10.1186/s12883-015-0304-6 (PMC4459447; doi:10.1186/s12883-015-0304-6)
Supplement: Additional file 4: — Pathological Diagnoses for Selected Participants. [file 12883_2015_304_MOESM4_ESM.docx]

**Additional File 4: Pathological Diagnoses for Selected Participants**

|  |  | AmnAD | AtAD |
| --- | --- | --- | --- |
| Pathological Diagnoses | | 25 | 8 |
| Alzheimer's Disease | Primary Diagnosis | 24 | 5 |
|  | Contributing Diagnosis | 1 | 3 |

**Additional File 4 Legend**: Pathological diagnoses are summarized for each clinical group. We conducted a post hoc chart review for participants with available pathological data; all individuals had Alzheimer’s disease (AD) pathology cited as a primary or major contributing factor which correlated with each patient’s clinical presentation. The individual in the amnestic Alzheimer’s disease (AmnAD) cohort with a contributing pathological diagnosis of AmnAD was pathologically diagnosed with chronic traumatic encephalopathy (clinically relevant AD plaques were found in the hippocampus and neurofibrillary tangles in limbic areas). The three individuals in the atypical Alzheimer’s disease (AtAD) cohort with a contributing diagnosis of AD were pathologically diagnosed with: 1) frontotemporal lobar degeneration TDP-43 Type B (clinically relevant AD plaques were found throughout the brain and neuritic plaques in the dorsal raphe and entorhinal cortex), 2) vascular brain injury (clinically relevant AD plaques and tangles were frequent in areas associated with memory functions), and 3) dementia with Lewy bodies (clinically relevant AD plaques and tangles were frequent in areas associated with memory functions). These data suggest that AD pathology was correctly recognized as a major contributor to patients’ clinical syndrome.
